# Alpha-synuclein Fibrils Inhibit Activation of the BDNF/ERK Signaling Loop in the mPFC to Induce Parkinson's Disease-like Alterations with Depression

**DOI:** 10.1007/s12264-024-01323-x

**Published:** 2024-11-28

**Authors:** Zhuoran Ma, Yan Xu, Piaopiao Lian, Yi Wu, Ke Liu, Zhaoyuan Zhang, Zhicheng Tang, Xiaoman Yang, Xuebing Cao

**Affiliations:** 1 Department of Neurology, Union Hospital, Tongji Medical College, Huazhong University of Science and Technology, Wuhan, 430000 China; 2 Department of Neurology, Renmin Hospital of Wuhan University, Wuhan, 430000 China

**Keywords:** Parkinson’s disease with depression, pS129, BDNF, ERK, CREB, mTOR, SEW2871

## Abstract

Depression (Dep) is one of the most common concomitant symptoms of Parkinson's disease (PD), but there is a lack of detailed pathologic evidence for the occurrence of PD-Dep. Currently, the management of symptoms from both conditions using conventional pharmacological interventions remains a formidable task. In this study, we found impaired activation of extracellular signal-related kinase (ERK), reduced levels of transcription and translation, and decreased expression of brain-derived neurotrophic factor (BDNF) in the medial prefrontal cortex (mPFC) of PD-Dep rats. We demonstrated that the abnormal phosphorylation of α-synuclein (pS129) induced tropomyosin-related kinase receptor type B (TrkB) retention at the neuronal cell membrane, leading to BDNF/TrkB signaling dysfunction. We chose SEW2871 as an ameliorator to upregulate ERK phosphorylation. The results showed that PD-Dep rats exhibited improvement in behavioral manifestations of PD and depression. In addition, a reduction in pS129 was accompanied by a restoration of the function of the BDNF/ERK signaling loop in the mPFC of PD-Dep rats.

**Supplementary Information:**

The online version contains supplementary material available at 10.1007/s12264-024-01323-x.

## Introduction

Depression (Dep) is one of the most common non-motor symptoms of Parkinson’s disease (PD), with an incidence of up to 40%. There is a consensus that depression significantly impairs the quality of life and cognitive function in patients with PD [1] and may even exacerbate motor symptoms [2]. The experimental evidence on the pathological mechanism of PD-Dep is limited, and the efficacy of antidepressant therapy varies considerably among PD-Dep patients. Selective 5-hydroxytryptamine reuptake inhibitors, such as sertraline, have been found to worsen the pre-existing tremor despite improving the mood in PD-Dep patients [3]. The efficacy of pramipexole, a traditional drug for PD, in treating depressive symptoms requires further validation through additional clinical trials [4, 5]. Therefore, our team endeavored to explore alternative pathological mechanisms of PD-Dep and provide innovative therapeutic concepts.

The characteristic pathological change in PD is the increased phosphorylation of Ser129 of alpha-synuclein (α-Syn; pS129) in the striatum and substantia nigra [6]. The SNCA gene encodes the α-Syn protein, and patients with clinically detected mutations in this gene often have early-onset non-motor symptoms such as depression [7]. Basic medical studies have demonstrated that mice overexpressing α-Syn exhibit a depressive-like phenotype at an early age [8]. Some researchers have found that the use of atorvastatin is able to reduce the expression of pS129 and improve depressive symptoms in PD patients [9]. These studies suggest a strong link between pS129 and PD-Dep, but the pathological mechanism by which pS129 is involved in the onset and progression of PD-Dep remains unclear.

α-Syn can diffuse within the brain [10]. The presence of pS129 in the prefrontal cortex (PFC) has been reported from clinical PD patients, the MPTP-induced PD monkey model, and PD rodent models [11–13]. A study of emotion reported that dysfunction of the medial PFC (mPFC) is associated with emotional deficits [14]. Volume reduction of the mPFC is one of the most common manifestations of the neurological abnormalities documented in major depressive disorder [15]. Xu *et al.* reported abnormalities within the mPFC using functional magnetic resonance imaging in depressed patients [16]. These findings suggest that the mPFC is an important region involved in depression. Chen *et al.* found that crocin improves synaptic plasticity in the mPFC and ameliorates depression-like behavior in a mouse with PD [17]. This suggests that the mPFC may be associated with comorbid depression in PD patients. However, the current studies not only lack direct evidence of a relationship between the mPFC and the onset of PD-Dep but also fail to explore the pathological changes caused by pS129 in the mPFC. Therefore, our team aimed to further investigate the underlying pathological mechanisms in the mPFC and provide a more precise reference target for the treatment of PD-Dep.

By binding to membrane tyrosine receptor kinase (TrkB), brain-derived neurotrophic factor (BDNF) induces endocytosis of TrkB to signal transduction and activates the downstream signaling pathway, ultimately leading to upregulating target gene expression. Clinical and basic science studies have confirmed a positive correlation between BDNF levels and antidepressant treatment efficacy [18, 19]. Downregulation of BDNF gene expression has been reported in the mPFC of the mouse model of depression [20]. BDNF deficiency has also been implicated in the pathogenesis of PD. A significant decrease in BDNF expression levels has been detected in the brains of PD patients [21] and the brains of α-Syn-overexpressing mice [8]. Our previous study showed that impaired BDNF/TrkB signaling is associated with PD [22]. However, there is limited evidence on whether the expression of BDNF is altered and regulated by α-Syn in the onset of PD-Dep. Based on these studies, we hypothesized that pS129 diffuses into the mPFC and induces the downregulation of BDNF expression, leading to the onset and progression of PD-Dep.

To test this hypothesis, we used preformed α-Syn fibrils (PFF) and stereotactically injected them into the right striatum of Sprague-Dawley rats. This method accurately simulated the pathological characteristics of diffuse pS129 in the brains of PD-Dep patients. Two months after the injection, behavioral and pathological markers corresponding to PD and depression were assessed in each group of rats. In addition, we applied transcriptome sequencing to tissue from the mPFC region, which was the focus of this study. We found that PFF rats exhibited behavioral changes associated with PD and depression, and pS129 overexpression was detected in both the substantia nigra and the mPFC. The sequencing results suggested that the expression of extracellular signal-regulated kinase (ERK) was altered, and this may be involved in the pathological mechanism of PD-Dep. We demonstrated that pS129 interferes with the normal physiological activity of the BDNF/ERK signaling pathway. In addition, we selected SEW2871 as a selective ERK agonist that can alleviate the pathological effect of pS129, ultimately providing novel evidence for the treatment of PD-Dep.

## Materials and Methods

### Preformed Alpha-synuclein Fibrils

The preformed alpha-synuclein fibrils used in the experiments were kindly provided by the Department of Neurology, Renmin Hospital of Wuhan University, Hubei, China. We did not generate unique reagents in this study.

## Animals

Sprague-Dawley rats were purchased from Liaoning Changsheng Biotechnology Co. The criteria for selecting rats were: male, 8 weeks old, weighing 240–300g before modeling. They were fed in the specific pathogen-free (SPF) animal laboratory of the Laboratory Animal Center, Huazhong University of Science and Technology. After allowing the rats to acclimate to the animal laboratory environment, we initiated the modeling process. Firstly, the rats were anesthetized with sodium pentobarbital (provided by an affiliated hospital experimental platform) at 0.1mL/50g and fixed on a digital stereotaxic apparatus. The fur between the ears was shaved, and sterilized with iodophor, and the skin was cut to expose the anterior and posterior fontanelles, which were adjusted to the same horizontal position, and the anterior fontanel was used as the origin to establish the coordinate axis. The right striatal location was as follows (in mm): anteroposterior +1.2, mediolateral −2.4, dorsoventral −4.0. As in our previous experiments, the right striatum was inoculated with 15 μg PFF or isovolumetric phosphate-buffered saline (PBS). The animals were then divided into two groups, PFF and Sham. The incision was sutured, and erythromycin ointment was applied. They were fed in the SPF laboratory for 2 months to allow completion of the model. We performed *in vivo* experiments as shown in Fig. S1.

## Primary Cultured PFC Neurons

To increase adhesion, the cell well plates were coated with 0.1mg/mL poly-L-lysine (Sigma, P1399) overnight in a 37℃ incubator, followed by two washes with purified sterile water. The well plates were dry when used. Mouse embryos (E16-18) were used for the primary neuron experiments *in vitro*. To minimize unwanted enzymatic digestion damage, we used 5-mL sterile tips to mechanically separate the neurons, which were centrifuged at 400 g for 3 min. The sediment was resuspended with the seeding medium (components shown below) and then incubated on plates at 37℃ under 5% CO_2_. We waited for the neurons to attach. After 4 h, the medium was replaced with a neuronal maintenance medium (components shown below) that facilitated the growth of neurons. We confirmed the growth conditions every day. The medium was exchanged at least once every 3 days. At 5 days *in vitro* (DIV5), PFF (2 μg/mL) or isovolumetric PBS was added to the medium and incubated for 48 h to allow for completion of the *in vitro* model (Fig. S1).

The seeding medium consisted of 39% Dulbecco’s modified Eagle’s medium/F12 (Gibco, C11330500BT) with 10% fetal bovine serum (Gibco, 10099141), and 1% penicillin/streptomycin (Gibco, 15140122); the neuronal maintenance medium consisted of 96% Neurobasal medium (Gibco, 10888022), 2% B-27 Supplement (Gibco, 17504044), 1% penicillin/streptomycin, and 1% GlutaMAX (Gibco, 35050061).

## Virus Transfection

At DIV3, primary PFC neurons were transfected with ERK-GFP-overexpressing lentiviral vectors (OE-Erk) and isovolumetric-only GFP lentiviral vectors (OE-NC) (Shanghai Genechem Co.), divided into an ERK over-expression group and a negative control group. After transfection for 2 days, stable expression of lentiviral GFP was assessed in neurons under a fluorescence microscope, followed by the addition of PFF (2 μg/mL) into the medium of both groups. After 2 days of incubation, tests were carried out.

## Treatment *In Vitro* and *In Vivo*

We applied a selective ERK activator (SEW2871, MCE, HY-W008947) as the ameliorator to upregulate ERK phosphorylation. SEW2871 was dissolved in 5% dimethyl sulfoxide and 95% saline. Both the *in vivo* and *in vitro* models were divided into three groups as follows: a negative control (PBS+solvent), a positive control (PFF+solvent), and a drug-treated group (PFF+SEW2871). Rats were given SEW2871 (0.5 mg/kg) or the isovolumetric solvent daily by intraperitoneal injection for 14 days after mode completion. Meanwhile, SEW2871 (1 μmol/L) or isovolumetric solvent was added to the primary PFC neurons and incubated for 48 h, after which tests were carried out.

## Behavioral Tests

*Adjusting Steps Test*: The rat was lifted by the base of the tail and the drive stem held away from the tabletop, allowing it to touch the tabletop with its forelimbs. The rat moved forward at ~1 m/4 s. During movement, the rat touched the tabletop with its forelimbs, and the number of forelimb touches was recorded. Separate recordings of the affected and healthy upper limbs were applied. The touch frequencies of the two sides were compared.

*Cylinder Test*: This test is used to assess a rat's forelimb preference in its exploratory activity. Each rat was placed in an upright transparent cylinder and allowed to explore the cylinder wall with their forelimbs. The number of times each rat touched the wall with the affected forelimb, the healthy forelimb, and both forelimbs were recorded over a 5-min period. The rats were not induced to stand and touch the cylinder wall during the experiment.

*Sucrose Preference Test*: Depressed animals may exhibit a reduced preference for sweet substances. Prior to the experiment, the animals underwent sucrose water acclimatization training, during which they were given 1% sucrose water to acclimate them to the substance. Solid food was removed before the start of the experiment and only sugar water and regular water were provided. We measured the consumption of both liquids periodically for 24 h. Upon completion of the experiment, we calculated the Sucrose Preference%, which represents the percentage of sugar water intake to total intake.

*Forced Swimming Test*: Experimental animals were placed in a confined space, creating an inescapably oppressive environment. We prepared an open cylindrical container free of other animal odors. Next, we added water at room temperature to the container and ensured that the rat could not escape from it. Each rat was placed in the cylinder and left for a few seconds until it was slightly acclimated to the water environment. The 6-min of forced swimming was started and recorded data included swimming time, climbing time, and resting time. The percentage of time in each of the three states was recorded.

*Novel Object Recognition Test*: Cognitive memory capacity was assessed by the behavioral approach of the time exploring familiar objects *versus* new unfamiliar objects. A quiet open field apparatus was prepared in advance to eliminate external stimuli. In the first round of experiments, two identical geometric objects were placed in the field and the rats were placed in the field to adapt to these objects for 6 min. The second round of experiments commenced after a 1-h interval. One of the old objects was replaced with a different new object and the rats were placed in the field again. The second round of experiments also lasted for 6 min, and the times of exploring the old and new objects were separately recorded. Before each rat entered the field, it was ensured that all odors left by the previous rat were eliminated. The recognition index is the ratio of the time of exploring the new object to the sum of the time of the old and the new objects.

## Enzyme-Linked Immunosorbent Assay

Based on the results of the preliminary experiments, it was necessary to quantify the BDNF in rats' mPFC tissue and primary PFC neurons. We used the BDNF ELISA Kit (Cusabio Co., CSB-E04504r).

## Protein Extraction

We added fresh brain tissue or primary PFC neurons to pre-cooled RIPA lysate and ground the samples in an ice bath, then left them on ice for 30 min. Tissue fractions were centrifuged at 4℃, 12,000 r/min, for 15 min to extract the supernatant. A BCA protein concentration assay kit (Boster, AR0146) was used to determine the concentration of the sample protein. Loading buffer 5× was added to the supernatant and the protein of the mixtures denatured at 100℃ (300 g for 5 min).

## Western Blot Assay

Each sample protein was subjected to electrophoresis using a PAGE Gel Rapid Preparation Kit (Epizyme Biomedical Technology Co., PG111-113) with quick high-resolution electrophoresis buffer (Servicebio, G2081-1L) at constant 200 V for 30 min, transferred to PVDF membranes (Millipore) with ice bath free quick transfer buffer (Servicebio, G2028-1L) at constant 400 mA, and blocked with quick blocking buffer (New Cell & Molecular Biotech Co., P30500). Next, membranes were incubated with matching primary antibodies dissolved in Universal Antibody Dilution Buffer (Abbkine, BMP4010) at 4℃ overnight and incubated with corresponding secondary antibodies at room temperature for 1 h. Last, the blots were developed using SuperKine™ West Femto Maximum Sensitivity Substrate (Abbkine, BMU102) and exposed on GeneGnome XRQ-NPC (Syngene). Images and data were analyzed using ImageJ and Graphpad Prism 9.0.

The antibodies used in western blots were as follows: TrkB (CST, Q16620), BDNF (Proteintech, 66292-1-Ig), Erk1/2 (Proteintech, 11257-1-AP), pErk1/2 (ABclonal, AP0974), α-synuclein (Abcam, ab212184), α-synuclein pSer129 (Abcam, ab51253), TH (Abcam, ab75875), DAT (Proteintech, 22524-1-AP), CREB (ABclonal, A11989), p-CREB (ABclonal, AP0903), mTOR (Proteintech, 66888-1-Ig), p-mTOR (Proteintech, 67778-1-Ig), 4EBP (GeneTex, GTX109162), p-4EBP (ABclonal, AP1334), Ubiquitin (Proteintech, 10201-2-AP), Ataxin-3 (ABclonal, A12992), and UBE2G2 (ABclonal, A10408). The above primary antibodies’ dilution was 1:1000. Internal reference antibodies were GAPDH (Abbkine, A01020) and β-tubulin (Abbkine, A01030) with 1:2000 dilution. The secondary antibodies were the corresponding-species HRP-conjugated antibodies with 1:10000 dilution.

## Extraction of Membrane Proteins

Neuronal membrane proteins were extracted using the Mem-PER Plus Membrane Protein Extraction Kit (ThermoFisher, 89842). Membrane protein extracts were subjected to downstream Western blot, which necessitated a modification in the internal reference antibody for ATP1A1 (Proteintech, 1:2000).

## Immunofluorescence

Brain tissue converted by the frozen-section method was delivered to Servicebio Co. for completion. The frozen sections were left at room temperature for 30 min, followed by permeabilization with 0.5% Triton X-100 for 30 min and blockade with 5% BSA for 1 h. Primary PFC neurons were cultured in 24-well plates with pre-placed cell slides and fixed with 4% paraformaldehyde. The permeabilizing and blocking steps were carried out for the frozen sections. Subsequently, both the frozen sections and neurons were incubated with primary antibodies at 4°C overnight. The following day, incubation with the secondary antibody was protected from light at room temperature for 1 h, followed by DAPI staining for 5 min. Finally, the sections and slides were mounted with an antifade mounting medium. Brain sections were scanned with VS120. Cell slides were photographed with FV3000. Data were analyzed using ImageJ and Graphpad Prism 9.0.

The antibodies used in immunofluorescence were as follows: α-synuclein pSer129 (Abcam, ab51253, 1:1000), TH (Abcam, ab75875, 1:200), TrkB (Proteintech, 13129-1-AP, 1:100), BDNF (Proteintech, 66292-1-Ig , 1:400), Erk1/2 (Proteintech, 66192-1-Ig, 1:800 & 11257-1-AP, 1:100), p-Erk1/2 (ABclonal, AP0974, 1:200), mTOR (Proteintech, 66888-1-Ig, 1:400), p-mTOR (Proteintech, 67778-1-Ig, 1:200). The fluorescent secondary antibodies were Alexa-fluor488, Alexa-fluor594 or cy3 (Abbkine) with 1:200 dilution.

## Immunochemical Staining

For immunohistochemical staining of frozen sections, the procedure was the same as for immunofluorescence staining until incubation with a secondary antibody. Sections were incubated with secondary antibody at room temperature for 1 h, protected from light, then stained with DAB (Servicebio, G1212-200T) for 5 min, and stained with Mayer's hematoxylin (Absin, abs9215). The sections were washed three times with PBS after each step. Brain sections were also scanned with VS120. Finally, the data is analyzed with ImageJ and GraphPad Prism 9.0.

The antibody used in the western blot was α-synuclein pSer129 (Abcam, ab51253, 1:1000). The secondary antibodies were the corresponding-species HRP-conjugated antibodies with 1:200 dilution.

## High-Performance Liquid Chromatography-Tandem Mass Spectrometry

Appropriate amounts of dopamine (Sigma, H8502), γ-aminobutyric acid (Sigma, A2129), and 5-hydroxytryptamine (Sigma, H9523) standard powders were weighed, dissolved into single standard stock solutions using methanol (Sigma, 439193) at 10 μg/mL, and stored at -20℃. Then the mixed standard solution was diluted with methanol to (ng/mL): 0.5, 1, 5, 10, 50, 100, 500, 1000, 5000, and 10,000. A subsequent test was carried out to plot the standard curve. 50% methanol was used as a solvent with 0.1% formic acid (Sigma, 159013) and 0.1% L-cysteine (Sigma, 168149) to form a methanol mixture. Samples were pulverized in liquid nitrogen and 1 mL of the methanol mixture was added. The sample methanol mixtures were vortexed together for 30 s, followed by sonication in an ice water bath for 20 min. Next, the mixture was centrifuged at 4℃ and 10,000 r/min for 10 min and the supernatant was isolated. We tested the sample supernatant on the Acquity UPLC H-Class Plus/Synapt XS after passage through a 0.22μmol/L filter membrane.

## Statistics Analysis

No statistical methods were applied to predetermine the sample size. Statistical tests used for each experiment are detailed in the figure legends. All data were analyzed using GraphPad Prism 9.0 software and a *P*-value <0.05 was considered statistically significant (ns: *P* ≥0.0.5; ∗*P* <0.05; ∗∗*P* <0.01; ∗∗∗*P* <0.001; ∗∗∗∗*P* <0.0001).

## Results

### PFF Induce PD Behavioral and Pathological Changes

After injection of α-synN103 PFF or an equal volume of PBS into the right striatum of rats which were then left for 2 months, we applied behavioral tests and detection of neuropathological changes associated with PD. We chose two commonly used behavioral tests for PD to assess the movement disorders of rats. In the cylinder test, rats in the PFF group not only lifted their forelimbs to touch the wall less frequently but also had a lower percentage of wall-touching by the contralateral forelimb than did the sham group (Fig. S2E). In the adjusting steps test, rats in the PFF group showed more evident limb incoordination (Fig. S2F). These behavioral results indicated that PFF-treated rats showed dyskinetic manifestations of PD after modeling for 2 months.

We next stained the striatum and substantia nigra pathological to assess whether PFF causes the typical pathologic changes of PD in rat brain regions. Compared with sham operation rats, the tyrosine hydroxylase (TH) signal in the ipsilateral substantia nigra was significantly decreased in the PFF group (Fig. S2A, B), and a high pS129 signal was also found (Fig. S2A, D). Basic medical findings have suggested that dopamine transporter protein (DAT), located on the presynaptic membrane of dopamine neurons, regulates synaptic gap dopamine concentration [23]. DAT imaging is a common clinical screening tool to support the diagnosis of PD [24]. Immunofluorescence staining for DAT was applied to the striatum of both rat groups, and as anticipated, the fluorescence signal in the striatum of the PFF group exhibited attenuation. (Fig. S2A, C).

From both behavioral and pathological perspectives, we demonstrated that α-synN103 PFF induce Parkinsonian changes in Sprague-Dawley rats.

## PFF Induce Depressive Behavioral Changes

We applied three behavioral tests to assess the level of depression in PFF model rats 2 months after they received stereotactic injections of equal volumes of PFF or PBS into the right striatum.

**Fig. 1:**
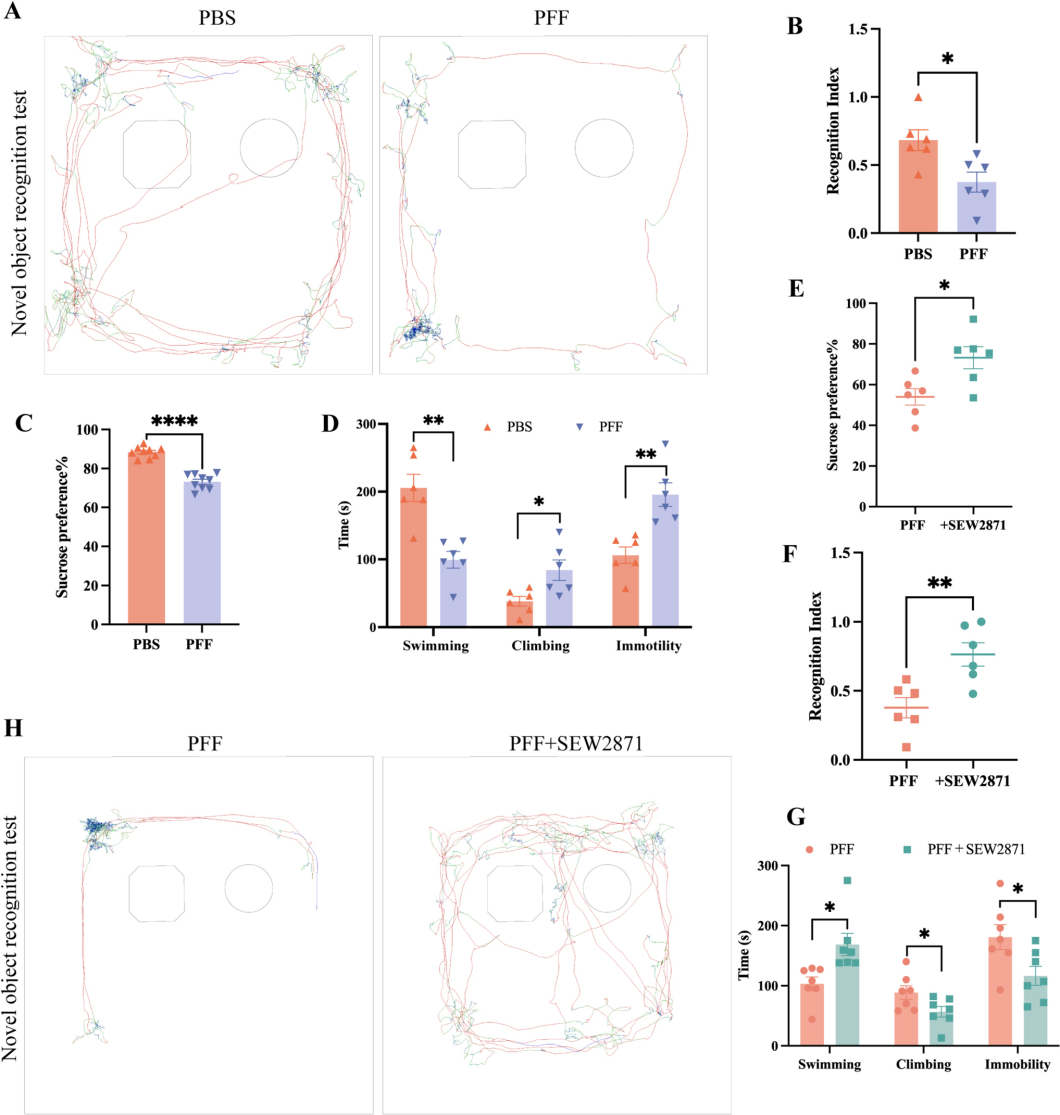
PFF induce depression-like behavioral changes in PD rats, whereas SEW2871 improves them to some extent. **A, H** Maps of representative trajectories in the novel object recognition test. **B**, **C** PFF make rats less interested in novelty or sweet soup. **D** PFF rats spend less time swimming and more time floating in pools. **E-G** SEW2871 ameliorates these behavioral deficiencies to a certain extent. In **B-G**, bars represent the mean, error bars represent the SEM, and symbols represent biologically independent replicates (*n*). *n =* 6. *P <0.05, **P <0.01, ***P <0.001.

Pleasure deprivation is one of the core manifestations of depression, and the sucrose preference test is a reliable behavioral test to assess pleasure deprivation in rodents [25, 26]. In comparison to the sham group, there was a significant decrease in the percentage of sucrose water consumption in PFF rats (Fig. 1C), illustrating that PFF rats had a reduced ability to experience pleasure. The forced swim test measures response ability in the face of acute unavoidable stress [27] and is widely used to assess antidepressant activity in rodents [28]. PFF rats maintained a floating state through slight hindlimb movements; In contrast, the rats in the sham group were able to swim back and forth between the bottom and surface of the container. Compared with the sham group, PFF rats exhibited decreased swimming time, increased resting time, and increased climbing time (Fig. 1D). These results suggested that PFF rats were more likely to fall into a state of despair and had low antidepressant activity when confronted with a high-pressure environment. Some studies have claimed that cognitive decline is common during and after depressive episodes in clinical patients [29, 30]. The new object recognition test in animal behavioral experiments is widely used to assess cognitive levels in rodents [31]. In this test, we found that rats in the PFF group not only had a smaller range of motion, but also spent less time moving (Fig. 1A), tended to be more sedentary in corners, and spent significantly less time exploring new objects (Fig. 1B). The implication of the above data was that the cognitive level of PFF rats has decreased.

Based on the above results, we can infer rats treated with α-SynN103 PFF, exhibited PD-related concurrent with depressive phenotypes and can accurately simulate the clinical progression of PD-dep patients. Furthermore, this implied that the abnormal over-expression of α-synuclein occurs in both the occurrence and progression of PD-Dep.

## PFF Trigger PD-like Pathology in the mPFC

**Fig. 2:**
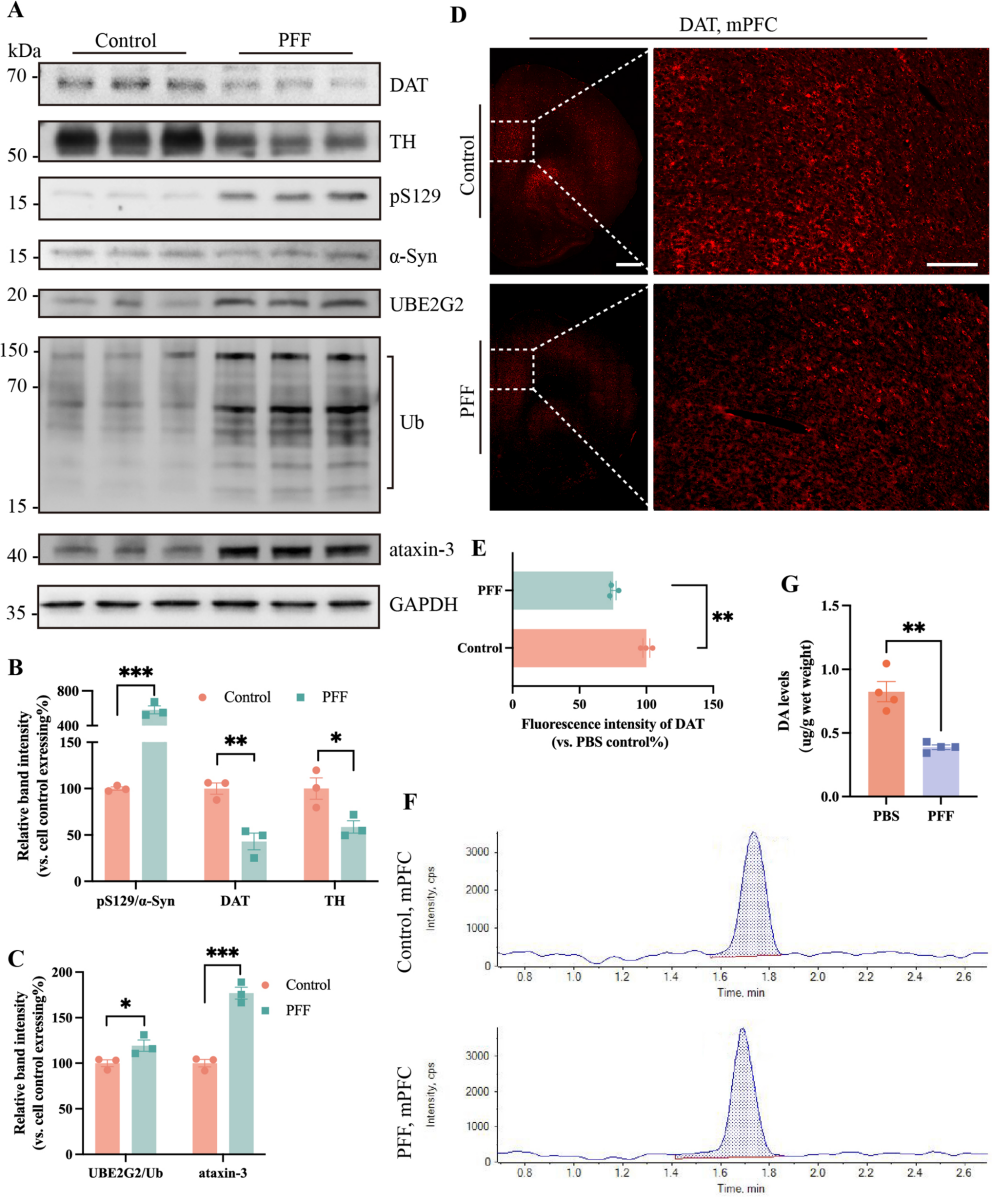
PFF triggers PD-like pathology in the ipsilateral mPFC.** A** Representative blots show a reduced dopaminergic phenotype and increased Lewy body-like inclusions in the mPFC. **B**, **C** The corresponding statistical analyses of **A**.** D** DAT is significantly reduced in the mPFC. **E** The corresponding statistical analyses of **D**. Scale bars, 1 mm (left) and 200 μm (right). **F**, **G** Representative HPLC-MS figures of DA levels. **B**, **C, E, G**, bars represent the mean, error bars represent the SEM, and symbols represent biologically independent replicates (*n*). *n =* 3. *P <0.05, **P <0.01, ***P <0.001.

Two months after the stereotaxic injection of PFF, we isolated the ipsilateral mPFC from rats with PD and depressive behavioral changes for RNA sequencing and analysis to determine whether the mPFC is involved in PD-Dep. We used gene set enrichment analysis (GSEA) to analyze the expression of PD gene profiles. Based on the heatmap results, it was evident that there was an upregulation in the expression of genes associated with PD pathology in the mPFC of PFF rats, and conversely, there was a significant downregulation in the expression of TH genes, which are the rate-limiting enzymes for dopamine anabolism (Fig. S3A). One study has reported that dopamine reuptake from synapses is accomplished by DAT, a process that is involved in emotion regulation in prefrontal regions [32]. We applied a western blot to the mPFC from the two groups, and the PFF group showed decreased expression of TH and DAT, with an increase of pS129 (Fig. 2A, B). We proceeded with the immunofluorescence staining of the mPFC for DAT and pS129. The DAT fluorescence intensity was diminished in the PFF group (Fig. 2D, E), while pS129 was significantly elevated (Fig. S3B, C). In addition, the expression of the SNCA gene, which encodes α-Syn, did not significantly differ between the two groups of rats as shown in GSEA (Fig. S3A). The western blot results also indicated no difference in non-phosphorylated α-Syn (Fig. 2A). The combined transcriptome and protein results confirmed that the main changes in the mPFC were an increase in phosphorylated Ser129 α-synuclein.

In addition to the above findings, we found that the expression of ubiquitin ligase E2 (UBE2G2), a gene associated with ubiquitination, was also moderately elevated in the PFF group. (Fig. S3A). The ubiquitin-proteasome system (UPS) regulates α-synuclein misfolding and aggregation [33], and patients with defective UPS genes develop a wide range of mixed brain proteinopathies, such as alpha-Syn, tau, and TDP-43 [34]. The levels of ubiquitin (Ub) and UBE2G2 were significantly elevated in the PFF group (Fig. 2A, C), indicating an active UPS in the mPFC. This raised the question as to why there was still pathological overexpression of pS129 in the mPFC. In a study on Machado-Joseph disease, ataxin-3 (ATX-3) was found to block the transfer of Ub from E2 ubiquitin ligase to E3 ubiquitin ligase, thereby disrupting the ubiquitin cascade reaction [35]. Not surprisingly, we found a high expression of ATX-3 in the mPFC of PFF rats (Fig. 2A, C). This finding accounted for the abnormal pS129 expression in the mPFC of PFF rats, despite the presence of a highly active UPS.

We subsequently isolated their prefrontal cortex and obtained primary neurons for *in vitro* culture. At DIV5, equal volumes of α-SynN103 PFF and PBS were each added to the neuronal medium and incubated for 48 h. The western blot results were consistent with those of animal tissues, with a significant decrease in DAT expression and an increase in pS129 (Fig. S3F-H). Confocal imaging demonstrated a marked increase in the fluorescence signal intensity of pS129 within the cytoplasm of primary neurons in the PFF group compared to that in the control group (Fig. S3D, E). These results from primary neurons further demonstrated that pS129 can induce PD-like pathological changes in mPFC neurons.

It has been discovered that the mPFC receives dopaminergic signaling inputs from other brain areas, and stimulation of these inputs has been shown to modulate synaptic plasticity and emotional processing in neurons of the mPFC [36], while dopamine depletion in the brain is the most common clinical manifestation of PD [37]. Therefore, we applied HPLC-MS to the mPFC of rats and confirmed a decrease in DA content of the PFF group (Fig. 2F, G).

Based on the results of *in vitro* and *in vivo* experiments, the mPFC of PFF rats showed increased pS129, which led to PD-like pathological alterations in neurons within this brain region.

## PFF Lead to Decreased Levels of BDNF *In Vivo* and *In Vitro*

In previous studies by our team, we found that BDNF/TrkB signaling is inhibited by α-Syn, triggering dopaminergic cell death in PD [38]. In another experiment, MPTP-model PD mice treated with metformin exhibited a decrease in pS129 with a concomitant increase in BDNF expression [39]. Furthermore, one study related to depression has demonstrated the critical role of BDNF in the survival, growth, and maintenance of neurons in key brain circuits involved in mood and cognitive functions [20, 39]. We speculated that there must be BDNF expression changes in the intersection of the pathological changes within PD and depression. However, further confirmation is needed to determine the expression level of BDNF in the mPFC at the onset of PD-Dep.

**Fig. 3:**
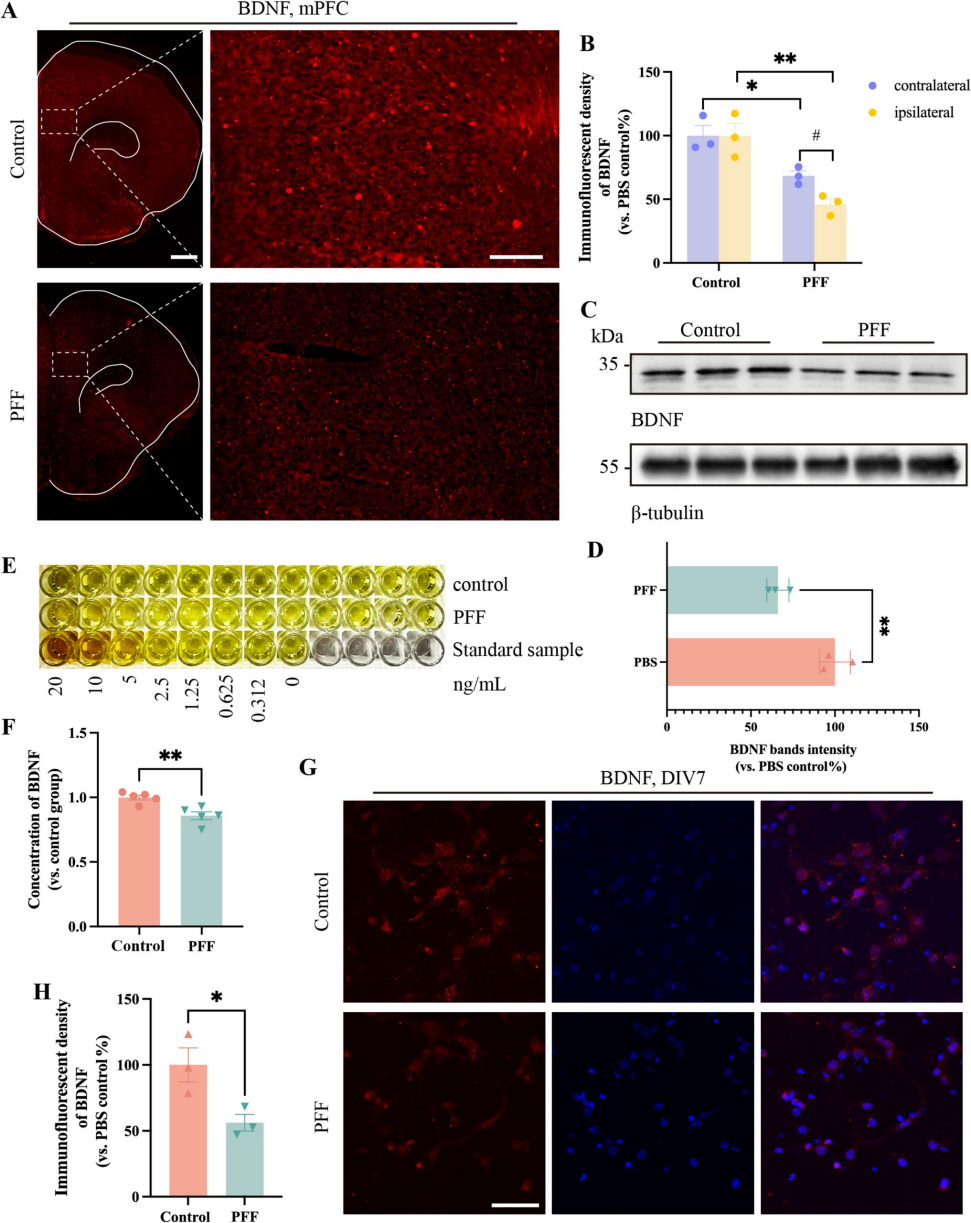
PFF lead to decreased levels of BDNF *in vivo* and *in vitro*. **A, G** Represent immunofluorescence images of BDNF in the mPFC and primary neurons. Scale bars, 1 mm (left) and 200 μm (right) in **A**; 50 μm in **G**. **C** Representative blots showing a reduced BDNF level compared to PBS controls in the ipsilateral mPFC. **E** ELISA test of BDNF uncovers a similar change in primary mPFC neurons. **B, D, F, H** The corresponding statistical analyses. Bars represent the mean, error bars represent the SEM, and symbols represent biologically independent replicates (*n*). *n =* 3 in **B** and **H**; 5 in **F**. *P <0.05, **P <0.01.

We applied immunofluorescence staining and western blot the mPFC in the PFF and sham groups. The results of both experiments showed that the expression of BDNF decreased (Fig. 3A-D). In addition, immunofluorescence staining revealed a reduced BDNF fluorescence signal in the primary neurons of the PFF group (Fig. 3G, H). BDNF is known to be a secreted neurotrophic factor that fulfills a role after binding to receptors through a variety of secretion modes [40]. To ensure the accuracy of our findings, we also performed ELISA for BDNF on primary neurons. The results were consistent with what was expected, showing a decrease in BDNF secretion in the PFF group (Fig. 3E, F).

Taken together, we demonstrated a decrease in BDNF content in the mPFC regions with abnormal pS129, which strongly suggests that down-regulation of BDNF is an important phenotypic outcome in the occurrence and progression of PD-Dep.

## BDNF/ERK Signaling Pathway is Inhibited in PD-Dep

There is a gap in the evidence from current studies on how the down-regulation of BDNF occurs in PD-Dep. As a result, clinical treatment regimens are often chosen to target a single disease with a lack of specificity, leading to many unintended side effects. The focus of our study was to identify key targets in the mPFC that regulate BDNF decline.

We searched for signaling pathways that may be involved in regulation through the results of RNA-Seq of mPFC tissue. A bubble diagram of the KEGG signaling pathway enrichment analysis demonstrated the potential involvement of numerous signaling pathways. We found that the pathway with the highest number of molecules involved is the MAPK signaling pathway (Fig. S4B). In the GSEA heatmap results of this pathway, we found a downregulation of the MAPK3 gene (Fig. S4A). It is well known that the mitogen kinase 3/1 gene (MAPK3/1) generates ERK1/2 upon transcription and translation [41]; it is one of the four family members of MAPK-mediated signaling cascade reactions (ERK1/2, JNK, p38, and ERK5). BDNF can affect many signaling pathways, among which ERK has been found to be a downstream signaling molecule for BDNF in depression research [42]. Studies on major depressive disorder have revealed disrupted ERK signaling and decreased BDNF content in the prefrontal cortex and hippocampus [43]. These findings imply that a decrease of BDNF expression in the mPFC in PD-Dep is highly likely to affect the ERK-mediated signaling pathway.

We compared the diversity of gene expression of molecules (BDNF, TrkB, ERK, MEK, Ras, CREB, mTOR, 4EBP, PI3K, Akt) that may be involved in the BDNF/ERK signaling pathway (Fig. S4D) and completed protein interaction network analysis (Fig. S4C). The results showed that altered protein phosphorylation is most likely the main way in which the BDNF/ERK signaling pathway is affected.

cAMP-response element binding protein (CREB) regulates gene transcription, and serine phosphorylation at position 133 (Ser133) plays an important role in CREB activity [44]. CREB is a downstream molecule of ERK [45]. Activation of ERK and CREB by phosphorylation leads to increased BDNF expression and exerts neuroprotective effects in Alzheimer's disease (AD) [46]. Based on the sequencing results from the mPFC, we hypothesized that phosphorylation activation of the ERK/CREB signaling pathway is inhibited in PD-Dep, leading to the downregulation of BDNF expression.

**Fig. 4:**
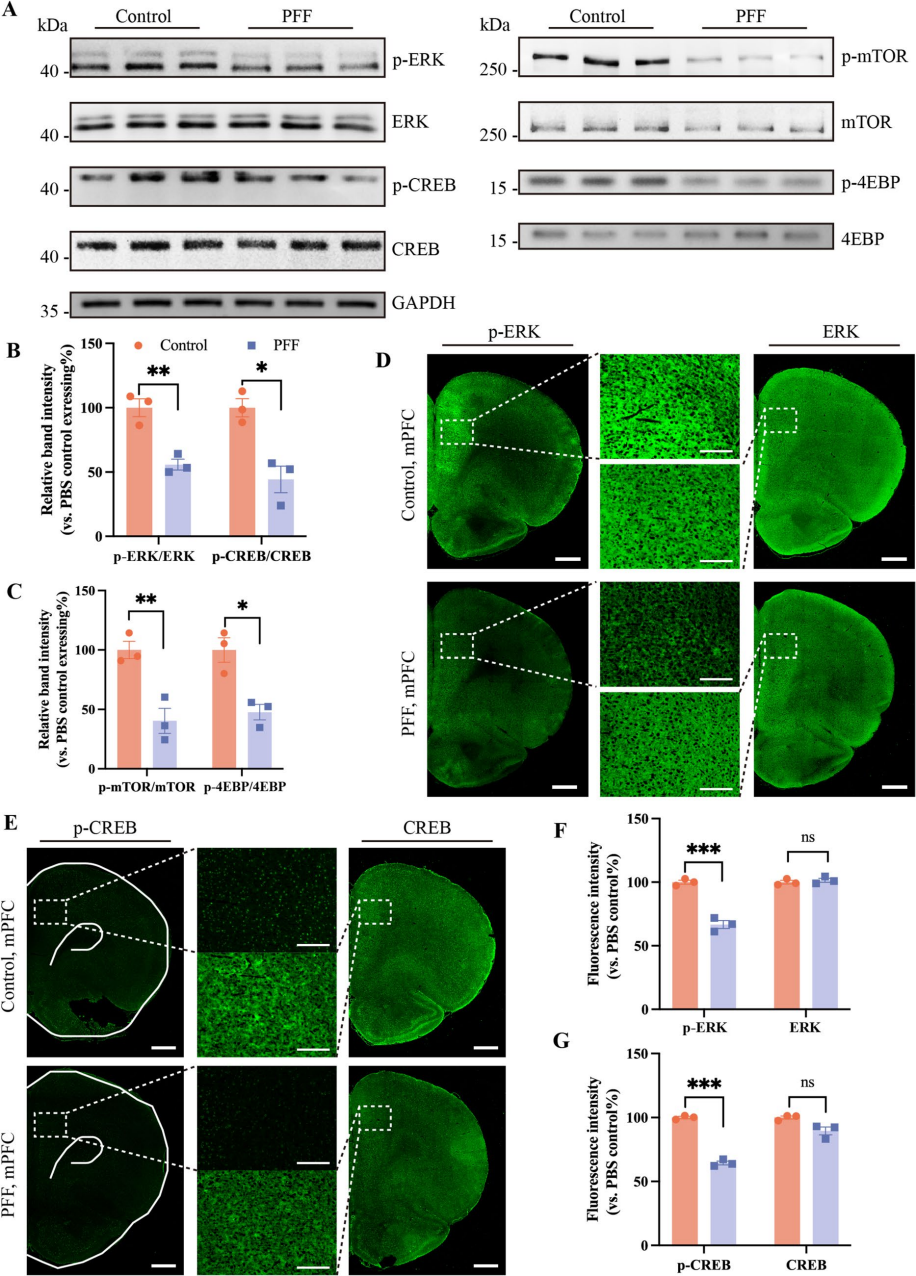
Reduced activation levels of ERK and its downstream proteins in the mPFC. **A** Representative blots showing downregulated levels of phosphorylation of ERK and its downstream proteins in the mPFC. **D**, **E** Represent immunofluorescence images of pERK/ERK and p-CREB/CREB in the ipsilateral mPFC. Scale bars, 1 mm (left and right) and 200 μm (middle). **B**, **C, F**, **G** The corresponding statistical analyses. Bars represent the mean, error bars represent the SEM, and symbols represent biologically independent replicates (*n*). *n =* 3. *P <0.05, **P <0.01, ***P <0.001.

To test the hypothesis, we performed a western blot assay, which showed that pERK and pCREB expression were significantly downregulated in the mPFC of the PFF group (Fig. 4A, B). The immunofluorescence results also showed a significant attenuation of the fluorescence intensity of phosphorylated proteins (Fig. 4D-G). Undoubtedly, the phosphorylation of the ERK/CREB signaling pathway occurred in the mPFC of PFF rats, and this also validated the results of our transcriptome sequencing results in the previous section.

CREB regulates the expression of target genes at the transcriptional level, but RNA still needs to be translated to form functioning proteins. Therefore, we set out to investigate whether the translation process was affected. Studies have reported that mRNA translation is tightly controlled by the mammalian target of rapamycin (mTOR) signaling and that mTOR-mediated translation is dependent on the regulation of eukaryotic translation initiation factor 4E binding protein (4EBP) [47–49]. In studies on the antidepressant effects of ketamine, investigators have used expression changes of BDNF and mTOR as indicators for assessing antidepressant effects [50, 51]. Given the above research, we determined whether mTOR and 4EBP were altered in PD-Dep. Western blot results showed that p-mTOR and p-4EBP in the mPFC of the PFF group also underwent significant down-regulation (Fig. 4A, C). These findings suggested that phosphorylation activation of proteins (mTOR and 4EBP) required for the translation process is restricted in mPFC neurons of PD-Dep rats.

In this section, we focused on exploring the pathological mechanisms that may lead to the down-regulated expression of BDNF in the mPFC of PD-Dep rats. Based on the RNA-Seq results, ERK was screened out as a key target, and protein phosphorylation restriction was the possible pathological mechanism process. The detection of phosphorylated CREB and mTOR/4EBP further demonstrated that limitations of transcription and translation occurred in neurons of the mPFC of PFF rats, ultimately leading to decreased expression of BDNF.

## PFF Limits the Internalization of TrkB at the Cell Membrane

We have previously discussed BDNF as the downstream molecule of ERK. However, it has also been demonstrated in many depression studies that BDNF can act as an upstream molecule of ERK, influencing its activation and downstream molecules [42]. This suggested a potential signaling loop between BDNF and ERK, by which they can mutually influence each other. In this section, we aimed to further explore the pathological mechanism of BDNF as the upstream molecule of ERK.

In a study on dendritic growth in cortical neurons, it was noted that after BDNF is bound to TrkB, TrkB is endocytosed into endosomes to continue signaling within the neuron [52]. Our previous findings have demonstrated that pS129 is bound to the structural domain of the kinase on TrkB, reducing its internalization and interfering with the signaling [38]. However, the mechanism by which pS129 interferes with BDNF/TrkB signals in PD-Dep has not yet been investigated. We hypothesized that, in PD-Dep, pS129 limits TrkB endocytosis, thereby contributing to the retention of TrkB on neuronal membranes and its failure to transmit signals to downstream ERK, ultimately leading to limited activation of ERK.

**Fig. 5:**
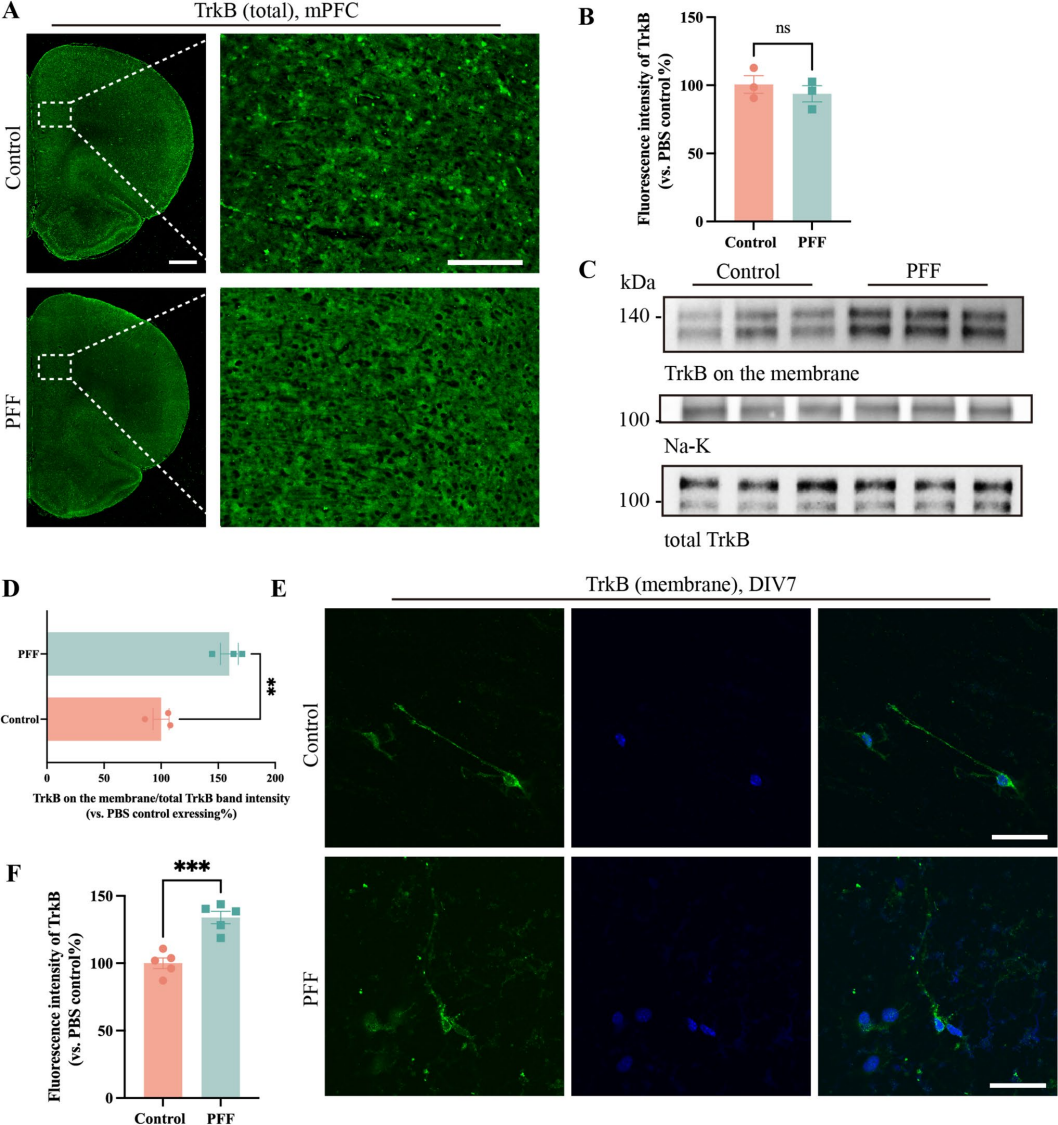
PFF limits the internalization of TrkB at the cell membrane. **A** No significant change in the total TrkB in the mPFC. Scale bar, 1mm (left) and 200 μm (right). **C** TrkB leaves more on the cell membrane after exogenous BDNF stimulation. TrkB bands show membrane (upper) and total (lower) levels. **E** TrkB is disorganized in axons and more remains at the cell membrane after stimulation by exogenous BDNF. Scale bars, 50 μm. **B, D, F** The corresponding statistical analyses. Bars represent the mean, error bars represent the SEM, and symbols represent biologically independent replicates (*n*). *n =* 3 in **B** and **F**; 5 in **D**. **P <0.01, ***P <0.001, ns = no significant difference.

To test the hypothesis, we initially quantified the total amount of TrkB molecules in the rat mPFC region. Immunofluorescence analysis revealed no difference in the fluorescence intensity of TrkB between the sham and PFF groups (Fig. 5A-C). Next, we extracted neuronal membrane proteins for western blots, revealing an increase in the content of TrkB on the membrane proteins of the PFF group, with no difference in the total TrkB content between the two groups (Fig. 5C, D). Subsequently, we applied immunofluorescence staining without membrane rupture. The fluorescence intensity of TrkB on the membrane was higher in the PFF group (Fig. 5E, F). These results indicated that pS129 leads to the retention of TrkB on neuronal membranes in the mPFC, resulting in a disruption of BDNF/TrkB signal transmission. As a downstream effector of BDNF, the activation of ERK was undoubtedly constrained.

In summary, pS129 participates in the BDNF/ERK signaling loop by inhibiting TrkB internalization and affecting the phosphorylation of other signaling molecules involved in the loop. This ultimately results in a negative feedback loop, with pS129 gradually increasing over time.

## Upregulation of ERK Restores BDNF Expression and Downregulates pS129 in Neurons.

We transfected primary PFC neurons with ERK-overexpressing lentivirus-controlled empty lentivirus and incubated them for 48 h. Subsequently, both groups of neurons were exposed to PFF in the medium for another 48 h. The western blots revealed that the high expression of pERK in neurons led to elevated levels of CREB phosphorylation (Fig. S5C, E) and significantly increased the expression of BDNF (Fig. S5C, D). Surprisingly, there was also a reduction in the level of pS129 to some extent (Fig. S5C, D). We then applied immunofluorescence co-staining. The results revealed that, along with the high expression of ERK in neurons, the co-localization of BDNF and ERK was increased, while conversely, the fluorescence signal of pS129 was decreased (Fig. S5A, B, F, G). These findings illustrate that lifting the restriction of ERK activation benefits the increase of BDNF expression while alleviating pS129 to some extent.

Although viral transfection methods are commonly used for basic experiments, they are difficult to perform for clinical treatment. Therefore, we decided to select drugs with high safety to increase ERK activation to improve PD-Dep.

SEW2871 is a widely used activator of sphingosine-1-phosphate receptors, commonly applied peripherally to improve vascular obstructive diseases or diseases with impaired barrier function [53, 54]. SEW2871 selectively activates ERK and promotes an increase in ERK phosphorylation [55]. In MPTP-induced mouse models of PD, SEW2871 has been reported to improve motor dyskinesia and the loss of dopaminergic neurons [56]. Furthermore, studies on AD have demonstrated that continuous intraperitoneal injection of SEW2871 for two weeks leads to improved cognitive function in AD rats [57] and significantly reduces Tau-Ser262 phosphorylation [58].

Based on the above research background, we hypothesize that SEW2871 can improve PD-Dep by promoting ERK phosphorylation. First, primary PFC neurons were cultured with PFF for 48 h and then treated with equal volumes of SEW2871 or solvent for 48 h. The western blot results revealed that neurons treated with SEW2871 exhibited elevated levels of ERK and CREB phosphorylation (Fig. S6E, F). In addition, there was an increase in the expression of BDNF and DAT, along with a decrease in pS129 (Fig. S6E, F). The immunofluorescence staining results revealed a significant increase in BDNF fluorescence following SEW2871 treatment (Fig. S6C, D).

These results demonstrated the potential of inducing ERK phosphorylation to improve PD-Dep. The *in vitro* results further supported our previous viewpoint that pS129 impacts the BDNF/ERK signaling pathway in the mPFC.

## SEW2871 Ameliorates the Down-regulated BDNF/ERK Signaling Pathway in the mPFC

Two months after all rats received stereotactic injections of PFF into the right striatum, they were divided into 2 groups. One group received SEW2871 treatment (SEW2871 group), while the other received an equal volume of solvent (PFF group). Both treatments were administered by continuous intraperitoneal injections for 14 days.

First, we applied behavioral tests. The rats in the SEW2871 group exhibited better limb coordination in the adjusting step test and the cylinder test than the rats in the PFF group (Fig. S2E, F), indicating that SEW2871 has a positive effect on the motor symptoms of PD. In the sucrose-preference test, the rats in the SEW2871 group showed an increased percentage of sucrose consumption (Fig. 1E). In addition, in the forced swimming test, the rats in the SEW2871 group swam for a longer duration and spent less time immobilized (Fig. 1G). Furthermore, in the new object recognition test, the rats in the SEW2871 group spent more time exploring the new object and had a higher recognition index than those in the PFF group (Fig. 1F, H). The results of these three behavioral tests also indicated that SEW2871 has a significant positive effect on depressive symptoms in PFF rats. In summary, SEW2871 has a synergistic effect in improving both types of symptoms in PD-Dep rats.

Next, we proceeded to assess the effect of the drug from a pathological perspective by staining the striatum and substantia nigra of rats. The results indicated that treatment with SEW2871 led to a reduction in the pS129 signal in the substantia nigra and upregulation of DAT signals in the striatum (Fig. S2A, C, D). However, there were no significant changes in the TH signal in the substantia nigra (Fig. S2A, B). These findings suggested that SEW2871 may have the potential to delay the pathological progression of PD to some extent.

**Fig. 6:**
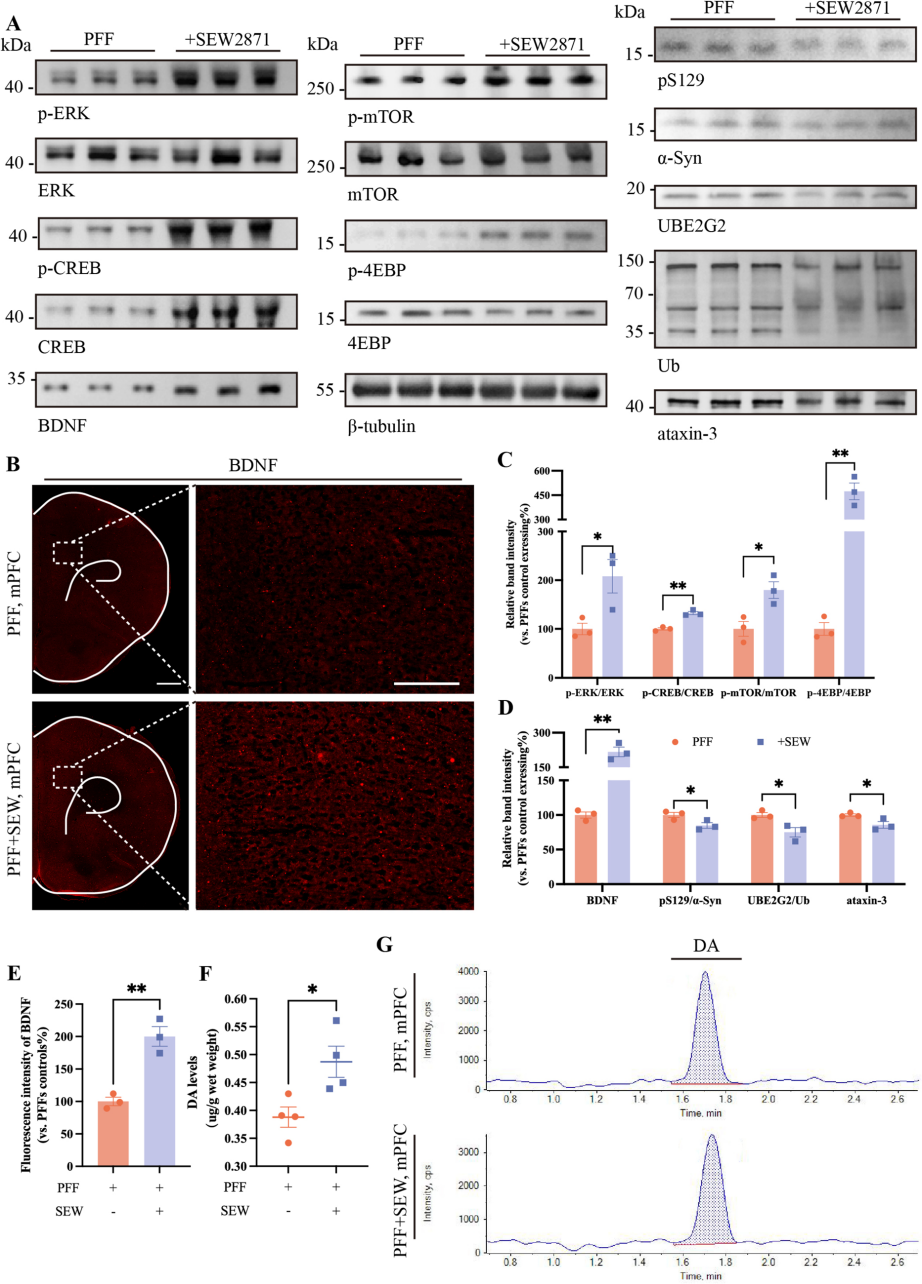
ERK agonist attenuates the abundance of inclusions and restores the decrease of BDNF and DA in the mPFC. **A** SEW2871 upregulates the levels of phosphorylation of ERK and its downstream proteins. The Lewy body-like inclusions showed the opposite alterations. **B** Represent immunofluorescence images showing a significant increase in BDNF. Scale bars, 1mm (left) and 200 μm (right). **G** SEW2871 somewhat mitigates the decrease in DA content. **C-F** The corresponding statistical analyses. Bars represent the mean, error bars represent the SEM, and symbols represent biologically independent replicates (*n*). *n =* 3 in **C-E**. *n =* 5 in **F**. *P <0.05, **P <0.01.

We applied immunofluorescence staining to the mPFC region and found that treatment with SEW2871 partially restored the expression levels of DAT (Fig. S6A, B) and BDNF (Fig. 6B, E), while the pS129 signal decreased (Fig. S6A, B). The western blot results demonstrated that BDNF levels increased, and pS129 levels decreased in the mPFC following SEW2871 exposure (Fig. 6A, D). In addition, the expressions of Ub, UBE2G2, and Ataxin-3, which are associated with the UPS, were found to be decreased (Fig. 6A, D). This suggested that pS129 effectively cleared up, and the overactive ubiquitination system returned to a normal state. We further applied HPLC-MS analysis to the mPFC. The results revealed a slight increase in DA levels in the SEW2871 group (Fig. 6F, G).

Furthermore, we tested the expression of ERK and its downstream signaling molecules. With SEW2871 treatment, pERK undoubtedly increased. There was a notable increase in the levels of pCREB, p-mTOR, and p-4EBP (Fig. 6A-C). These findings suggested that the limited phosphorylation of ERK and downstream molecules involved in the regulation of transcription and translation in the mPFC were restored by SEW2871.

These data are sufficient to demonstrate that SEW2871 has the potential to remove pS129, restore the activation-restricted BDNF/ERK signaling pathway, and ultimately confer neuroprotective effects on the mPFC region. In addition, SEW2871 has positive effects on motor impairment and depressive symptoms in rats.

## Discussion

Previous studies have used the rifampicin model to simulate PD with depression. Chronic administration of rifampicin has been shown to elicit depressive behaviors in rodents, as well as induce striatal dopamine depletion and some pathological processes associated with PD [59]. However, the typical pathological hallmark in clinical PD patients is the aberrant expression and accumulation of pS129. Therefore, in exploring the potential mechanisms of PD-Dep, our primary concern lies in understanding the pathologic mechanism of action of pS129. There appears to be a limited correlation between the reserpine model and pS129. Studies have shown that α-Syn PFF can serve as "seeds" that prompt the aggregation of endogenous α-Syn into insoluble Lewy bodies [60]. Asparagine endopeptidase cleaves at the N103 of α-Syn and exerts a "seeding" action that triggers α-Syn aggregation and leads to dopaminergic neuronal degeneration [61]. In this study, the α-Syn N103 PFF model rats exhibited altered behavior associated with PD and depression, along with loss of dopaminergic neurons and pS129 in the substantia nigra. Based on these data, we believe that the PFF model offered more advantages for studying the pathological mechanisms of PD-Dep.

pS129 can diffuse through neuronal axonal transport to brain regions other than the striatum and substantia nigra [62]. Our experimental results demonstrated that the upregulation of pS129 and the downregulation of TH, DAT, and BDNF occurred in the mPFC of PFF rats. These indicated that a series of pathological alterations initiated by the diffusion of pS129 into the mPFC region may play an important role in the onset and progression of PD-Dep.

The RNA-Seq results from the mPFC showed a significant alteration in the MAPK signaling pathway and a downregulation of the ERK gene. Furthermore, the analysis of protein interactions revealed significant changes in protein phosphorylation within the mPFC. The decrease in pERK levels in the mPFC suggested that ERK activation is disrupted in the mPFC of PFF rats. The ERK/CREB pathway regulates transcription [45], while the ERK/mTOR/4EBP pathway regulates translation [63]. Both are influenced by ERK phosphorylation. In the mPFC of the PFF group, the phosphorylation levels of CREB and mTOR were decreased. These findings indicate that both transcriptional and translational processes are constrained in the mPFC of PFF rats, ultimately resulting in the downregulation of target gene expression. BDNF is a target gene and downstream molecule of these signaling pathways [52, 64–66]. Thus, we posited that limited phosphorylation of ERK in the mPFC influences the activation of various downstream molecules involved in transcription and translation processes, ultimately leading to a decrease in BDNF gene expression.

pS129 interferes with TrkB internalization and hinders BDNF/TrkB signal transduction [38]. In addition, ERK may also serve as a downstream effector of BDNF [42]. Our results confirmed that there was no alteration in total TrkB expression, but an increase in neuronal membrane-bound TrkB levels. This suggests that phosphorylated ERK regulates the expression of the target gene BDNF, which in turn influences ERK activation, creating a feedback loop. However, the heightened pS129 anchored TrkB to the membrane and disrupted the BDNF/TrkB signaling pathway, resulting in activation barriers for all the signaling molecules involved. Ultimately, the signaling loop has a cumulative detrimental impact.

ERK represents the convergence of transcription and translation processes. Consequently, we chose to intervene in ERK with the objective of restoring the normal functioning of the above signaling loops. The primary neurons with transfected ERK-overexpressing lentiviruses demonstrated resistance to the downregulation of activation of the BDNF/ERK signaling loop caused by PFF. Given the extensive biological activities associated with ERK, it was deemed inadvisable to utilize drugs that specifically target ERK. Instead, we chose SEW2871, a selective ERK activator that is capable of crossing the blood-brain barrier (BBB). Our results demonstrated that PFF rats treated with SEW2871 exhibited improved behavioral performance associated with PD and depression. Furthermore, there was a decrease in pS129 in the substantia nigra and mPFC. In addition, following treatment with SEW2871, ERK phosphorylation was upregulated in the mPFC, leading to restoration of neuronal transcription and translation. This was concomitant with elevated expression levels of BDNF and partial restoration of dopamine expression.

Many non-neuronal cells, such as astrocytes and microglia, are also present in the mPFC. These cells are closely associated with autophagy and inflammation, and they mediate the deposition of α-Syn. The RNA-Seq results from the mPFC showed high expression of the LRRK2 gene associated with autophagy in the PFF group, indicating active autophagy in the mPFC. We speculate that non-neuronal cells in the mPFC may have also undergone pathological changes due to the diffusion of α-Syn.

SEW2871 has many advantages as a therapeutic agent. Firstly, its oral administration has been shown to be safe and can improve colitis in mice [67], which means that SEW2871 is more suitable for clinical translation in the form of oral drugs. Secondly, previous research has demonstrated the safety and therapeutic benefits of SEW2871 in both PD [56] and AD [57, 58], suggesting that it has the potential to improve neurodegenerative diseases. Finally, our findings support the conclusion that SEW2871 can benefit both the motor and depressive symptoms in PD. However, we also recognize that SEW2871 has some limitations as a therapeutic agent, which need to be further improved and expanded. The potential of SEW2871 to regulate vascular integrity following activation of S1PR1 has been demonstrated [68]. Due to the disruption of BBB integrity in epileptic patients, the use of SEW2871 increases leakage and induces a worsening of epilepsy [69]. In addition, PD-Dep patients at risk of cerebral hemorrhage are also excluded from treatment with SEW2871. Based on the above discussion it can be inferred that the use of SEW2871 is inappropriate if PD-Dep patients have concomitant diseases associated with disruption of vascular integrity. There have been many studies utilizing Au nanoparticles for the treatment of neurological disorders [70–72]. This treatment bypasses the BBB, reducing the possibility of damaging the BBB and vascular integrity. We plan to update the treatment modality through nanoparticles in future studies, allowing for a wider range of drug applicability. Although we used the PFF rat model to approximate the pathophysiological state of PD patients as closely as possible, there are undeniable differences between animal models and human disease. In the future, we plan to enrich the variety of animal models, such as mice, monkeys, and other species to test the efficacy and tolerance of SEW2871.

## Conclusion

**Fig. 7:**
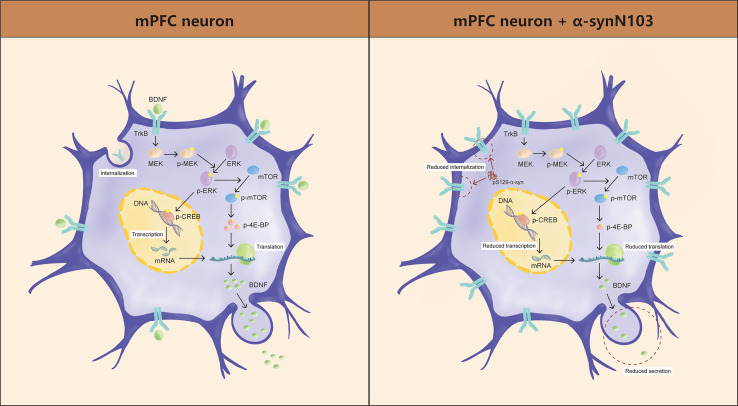
Graphical abstract of the onset of PD-Dep. Left, normal condition in mPFC neurons; right, pathological action of pS129 in mPFC neurons.

We propose that the BDNF/ERK signal loop disturbed by pS129 is the key pathological mechanism underlying the onset and progression of PD-Dep. Through the targeted activation of ERK, SEW2871 can counteract the pathological effect of pS129, normalize BDNF expression in the mPFC, and reduce the level of pS129 in the substantia nigra and mPFC region. This intervention ultimately leads to symptom improvement for both diseases, highlighting the potential clinical translation of this approach. The pathological process of PD-Dep is shown in Figure 7.

## Supplementary Information

Below is the link to the electronic supplementary material.

Supplementary file1 (PDF 1673 kb)

## Data Availability

Requests for resources of all data generated in this study should be directed to the lead contact (dryoung94@163.com).
