# Supplementary material for: Alpha-synuclein Fibrils Inhibit Activation of the BDNF/ERK Signaling Loop in the mPFC to Induce Parkinson's Disease-like Alterations with Depression
Source: Neurosci Bull. 2024 Nov 28;41(6):951–69. doi: 10.1007/s12264-024-01323-x (PMC12158912; doi:10.1007/s12264-024-01323-x)
Supplement: Supplementary file 1 — Supplementary file1 (PDF 1673 kb) [file 12264_2024_1323_MOESM1_ESM.pdf]

Supplemental Figures Legends

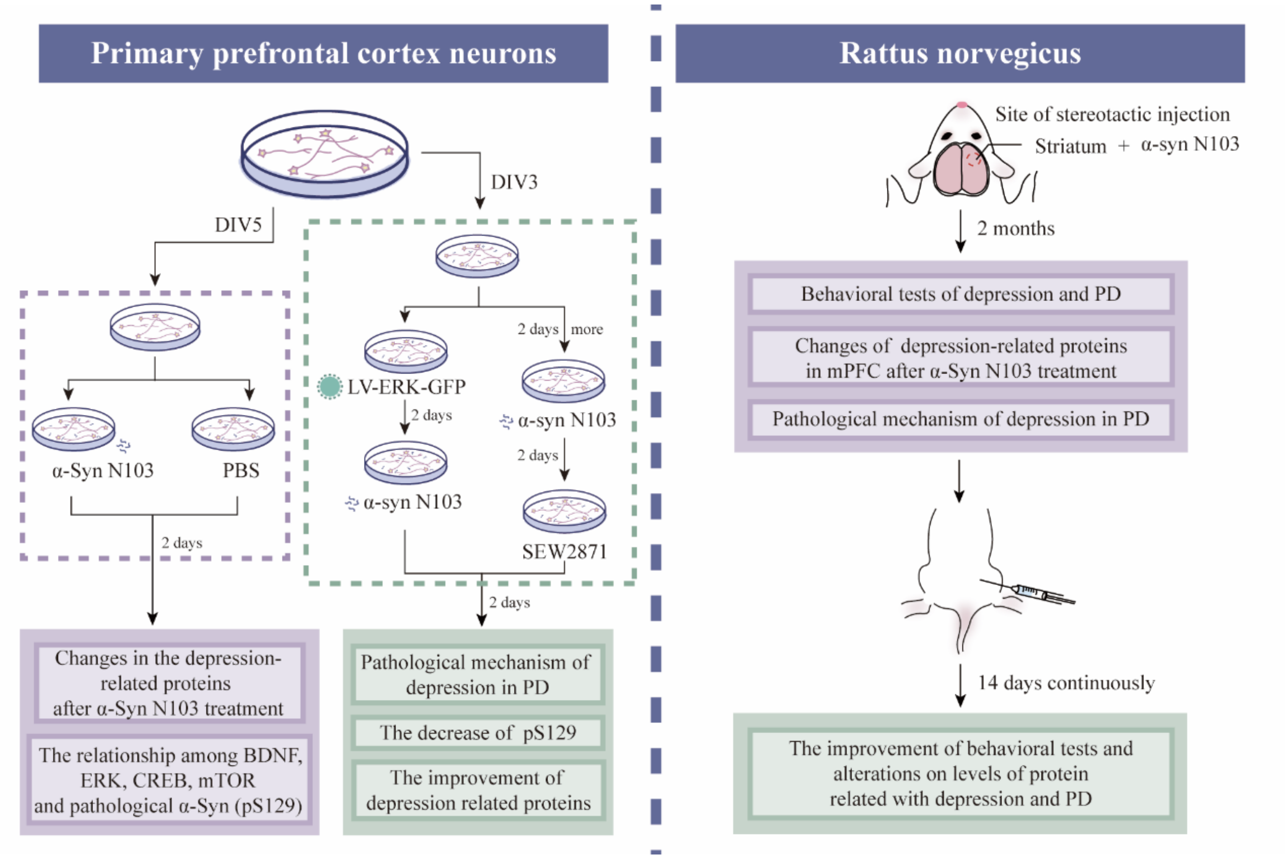

Fig. S1 Experiments followed the process shown above. **Left:** Cortical neurons are collected from mouse E18 embryos. PFF are added 5 days after cell culture and left for 2 days before drug intervention. Depending on the need, constructed lentivirus was added 48 h before PFF treatment. **Right:** Either PBS vehicle or α-SynN103 PFF were injected into the striatum. Behavioral tests were applied 2 months after injection. SEW2871, the ERK agonist, was injected intraperitoneally for 14 consecutive days if needed.

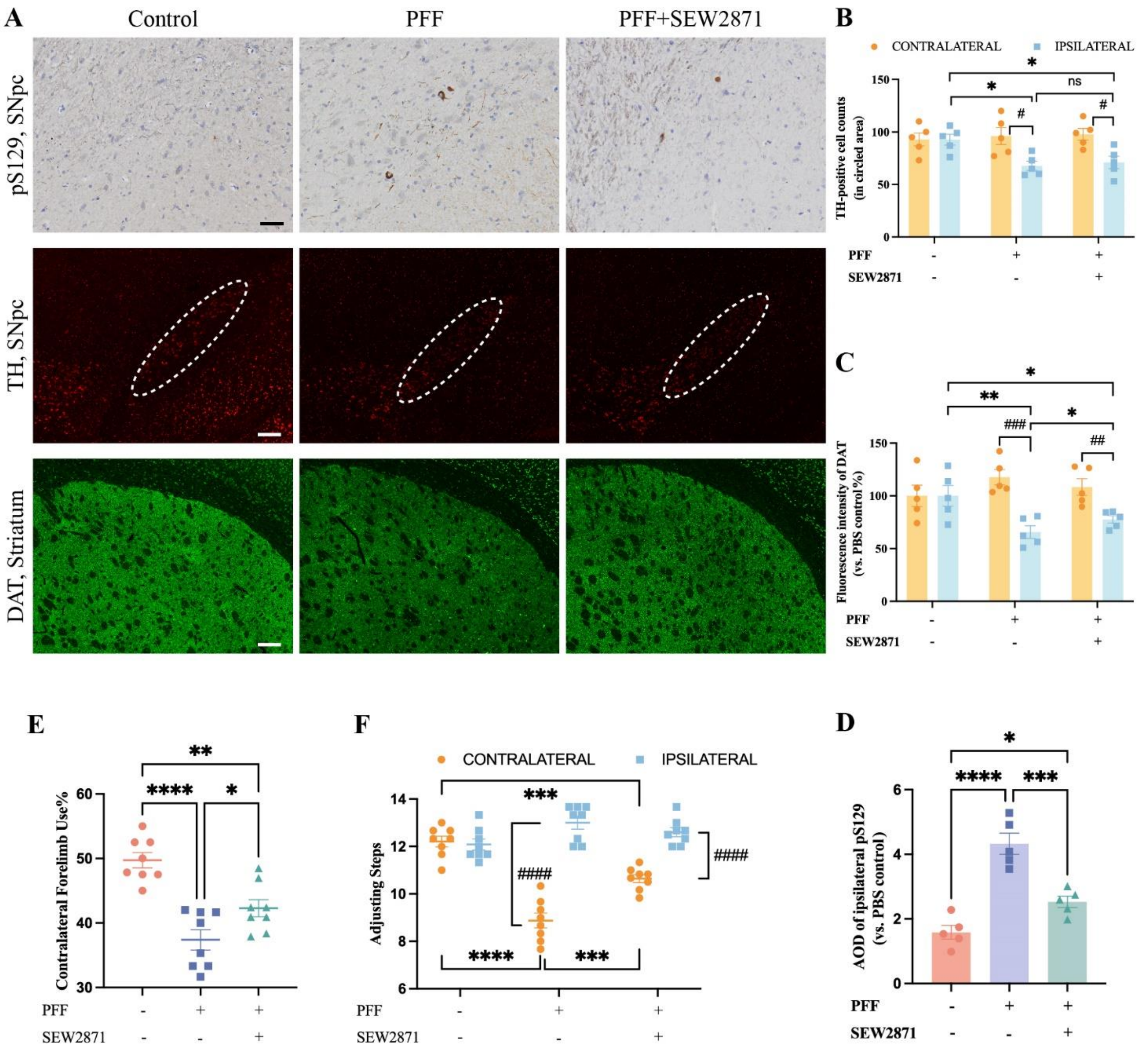

Fig. S2 The administration of ERK agonists slightly ameliorates the behavioral changes and degenerative alterations in nigrostriatal dopamine neurons that are induced by PFF in rats. **A** Representative immunohistochemical images of pS129 and immunofluorescence images of TH and DAT. Scale bars, 50 μm (in IHC images) and 200 μm (in IF images). **B-D** The corresponding statistical analyses of immunostaining. $n = 5$. $^{*}P < 0.05$, $^{**}P < 0.01$. **E**, **F** The PFF rats exhibit a reduced use of the contralateral limb. The administration of ERK agonists has a somewhat ameliorating effect on limb incoordination. $n = 8$. $^{*}P < 0.05$, $^{**}P < 0.01$, $^{***}P < 0.001$, $^{****}P < 0.0001$. Bars represent the mean, error bars represent the SEM, and symbols represent biologically independent replicates (n).

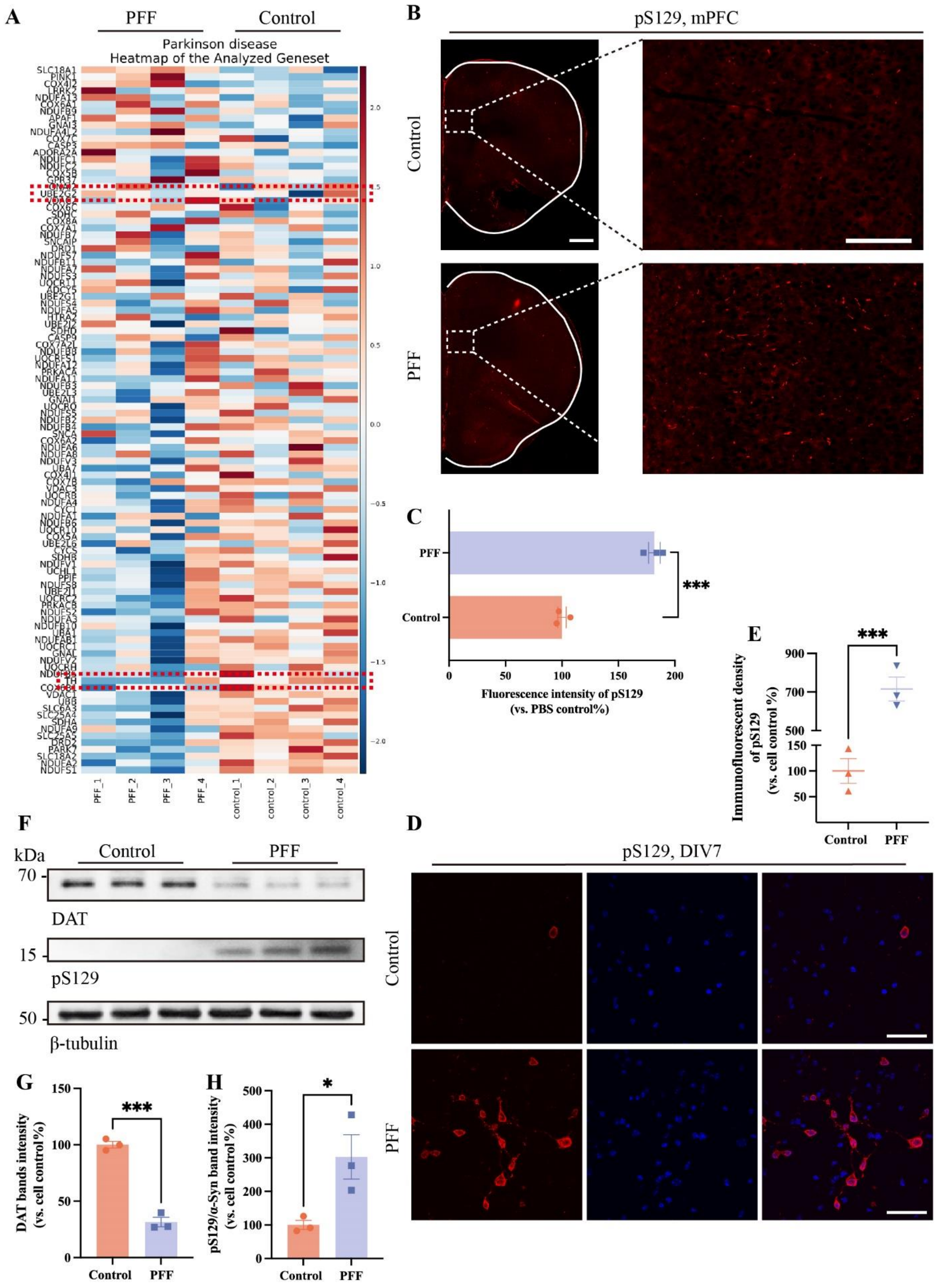

Fig. S3 Alteration of S129 in mPFC neurons. **A** GSEA is an expression data set sorted by correlation

with PD and the corresponding heat map. **B** Representative immunofluorescence images of pathological inclusions in the mPFC. Scale bars, 1 mm (left) and 200 μm (right). **D, F** DAT is significantly reduced, and levels of pS129 are increased in PFF-treated neurons. Scale bars, 50 μm in **D**. **C, E, G**, **H** The corresponding statistical analyses. Bars represent the mean, error bars represent the SEM, and symbols represent biologically independent replicates (n). $n = 3$. *P <0.05, ***P <0.001.

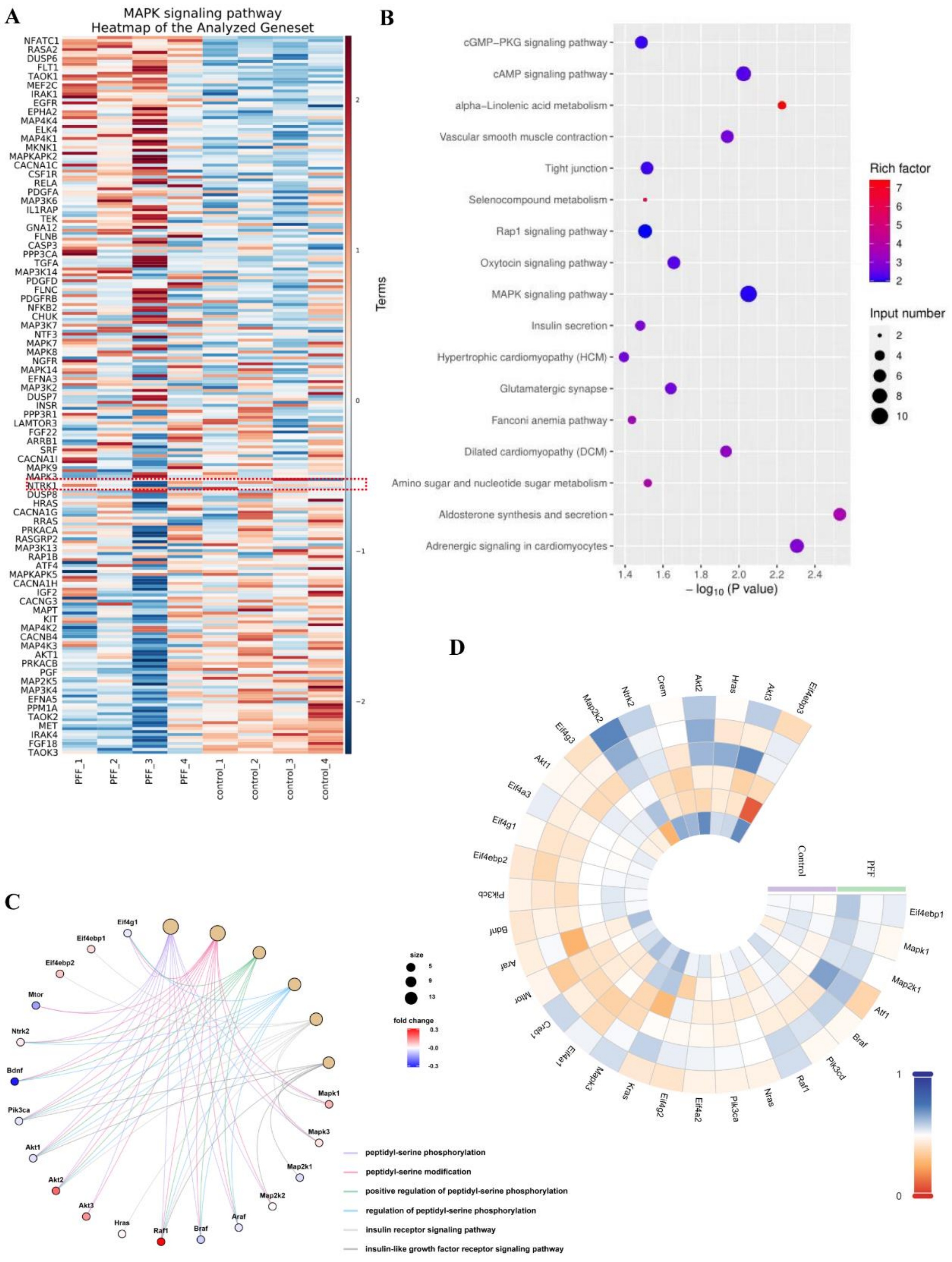
A
MAPK signaling pathway
Heatmap of the Analyzed Geneset
NFATC1
RASA2
DUSP6
FLT1
TAOK1
MEF2C
IRAK1
EGFR
EPHA2
MAP4K4
ELK4
MAP4K1
MKNK1
MAPKAPK2
CACNA1C
CSF1R
RELA
PDGFA
MAP3K6
IL1RAP
TEK
GNA12
FLNB
CASP3
PPP3CA
TGFA
MAP3K14
PDGFD
FLNC
PDGFRB
NFKB2
CHUK
MAP3K7
NTF3
MAPK7
MAPK8
NGFR
MAPK14
EFNA3
MAP3K2
DUSP7
INSR
PPP3R1
LAMTOR3
FGF22
ARRB1
SRF
CACNA1I
MAPK9
MAPK3
NTRK1
DUSP8
HRAS
CACNA1G
RRAS
PRKACA
RASGRP2
MAP3K13
RAP1B
ATF4
MAPKAPK5
CACNA1H
IGF2
CACNG3
MAPT
KIT
MAP4K2
CACNB4
MAP4K3
AKT1
PRKACB
PGF
MAP2K5
MAP3K4
EFNA5
PPM1A
TAOK2
MET
IRAK4
FGF18
TAOK3
PFF_1
PFF_2
PFF_3
PFF_4
control_1
control_2
control_3
control_4
2
1
0
−1
−2
Terms
B
cGMP−PKG signaling pathway
cAMP signaling pathway
alpha−Linolenic acid metabolism
Vascular smooth muscle contraction
Tight junction
Selenocompound metabolism
Rap1 signaling pathway
Oxytocin signaling pathway
MAPK signaling pathway
Insulin secretion
Hypertrophic cardiomyopathy (HCM)
Glutamatergic synapse
Fanconi anemia pathway
Dilated cardiomyopathy (DCM)
Amino sugar and nucleotide sugar metabolism
Aldosterone synthesis and secretion
Adrenergic signaling in cardiomyocytes
1.4
1.6
1.8
2.0
2.2
2.4
− log10 (P value)
Rich factor
7
6
5
4
3
2
Input number
2
4
6
8
10
C
Eif4g1
Eif4ebp1
Eif4ebp2
Mtor
Ntrk2
Bdnf
Pik3ca
Akt1
Akt2
Akt3
Hras
Raf1
Braf
Araf
Map2k2
Map2k1
Mapk3
Mapk1
size
5
9
13
fold change
0.3
-0.0
-0.3
peptidyl-serine phosphorylation
peptidyl-serine modification
positive regulation of peptidyl-serine phosphorylation
regulation of peptidyl-serine phosphorylation
insulin receptor signaling pathway
insulin-like growth factor receptor signaling pathway
D
Map2k2
Ntrk2
Crem
Akt2
Hras
Akt3
Eif4ebp3
Eif4g3
Akt1
Eif4a3
Eif4g1
Eif4ebp2
Pik3cb
Bdnf
Araf
Mtor
Creb1
Elk1
Mapk3
Kras
Eif4g2
Eif4a2
Pik3ca
Nras
Raf1
Pik3cd
Braf
Atf1
Map2k1
Mapk1
Eif4ebp1
Control
PFF
1
0

Fig. S4 Representative bioinformatics analyses. **A** GSEA: an expression data set sorted by correlation with the MAPK signaling pathway and the corresponding heat map. **B** Bubble diagram of KEGG signaling pathway enrichment analysis. **C** Screening gene interaction network map. **D** Screening gene expression differential loop

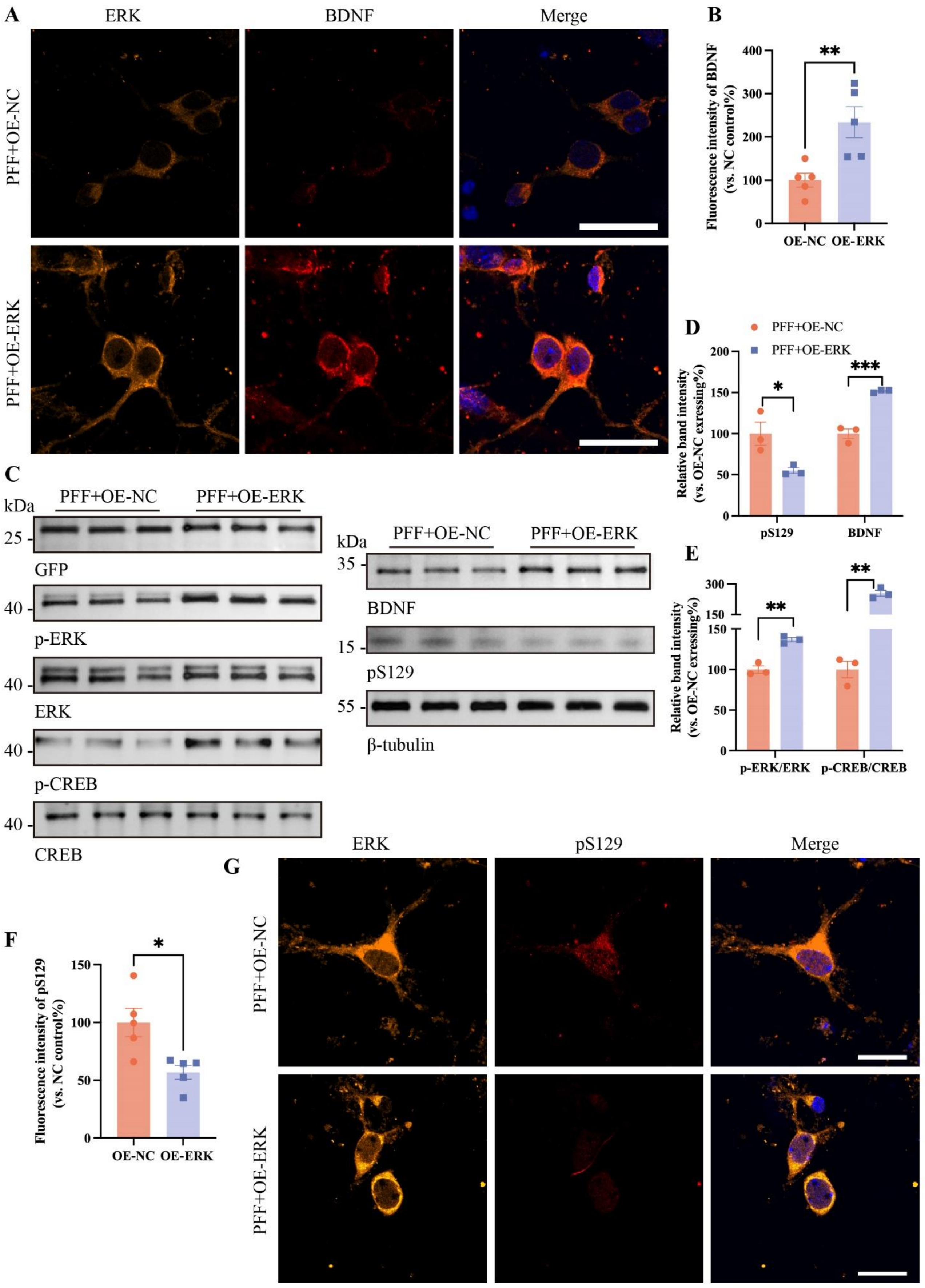
A
ERK
BDNF
Merge
PFF+OE-NC
PFF+OE-ERK
B
Fluorescence intensity of BDNF (vs. NC control%)
**
OE-NC
OE-ERK
C
kDa
PFF+OE-NC
PFF+OE-ERK
25
GFP
40
p-ERK
40
ERK
40
p-CREB
40
CREB
35
BDNF
15
pS129
55
β-tubulin
D
PFF+OE-NC
PFF+OE-ERK
Relative band intensity (vs. OE-NC exressing%)
*
***
pS129
BDNF
E
Relative band intensity (vs. OE-NC exressing%)
**
**
p-ERK/ERK
p-CREB/CREB
F
Fluorescence intensity of pS129 (vs. NC control%)
*
OE-NC
OE-ERK
G
ERK
pS129
Merge
PFF+OE-NC
PFF+OE-ERK

Fig. S5 Overexpression of ERK promoter BDNF expression and ameliorates PD-like pathology. **A, G** Representative images of BDNF (**A**) or pS129 (**G**) co-stained with ERK1/2 in the mPFC. Scale bar, 50 μm. **C** Representative blots of ERK/CREB/BDNF and pS129. **B, D-F** The corresponding statistical analyses. Bars represent the mean, error bars represent the SEM, and symbols represent biologically independent replicates (n). n = 5 in **B** and **F**. n = 3 in **D** and **E**. *P <0.05, **P <0.01, ***P <0.001.

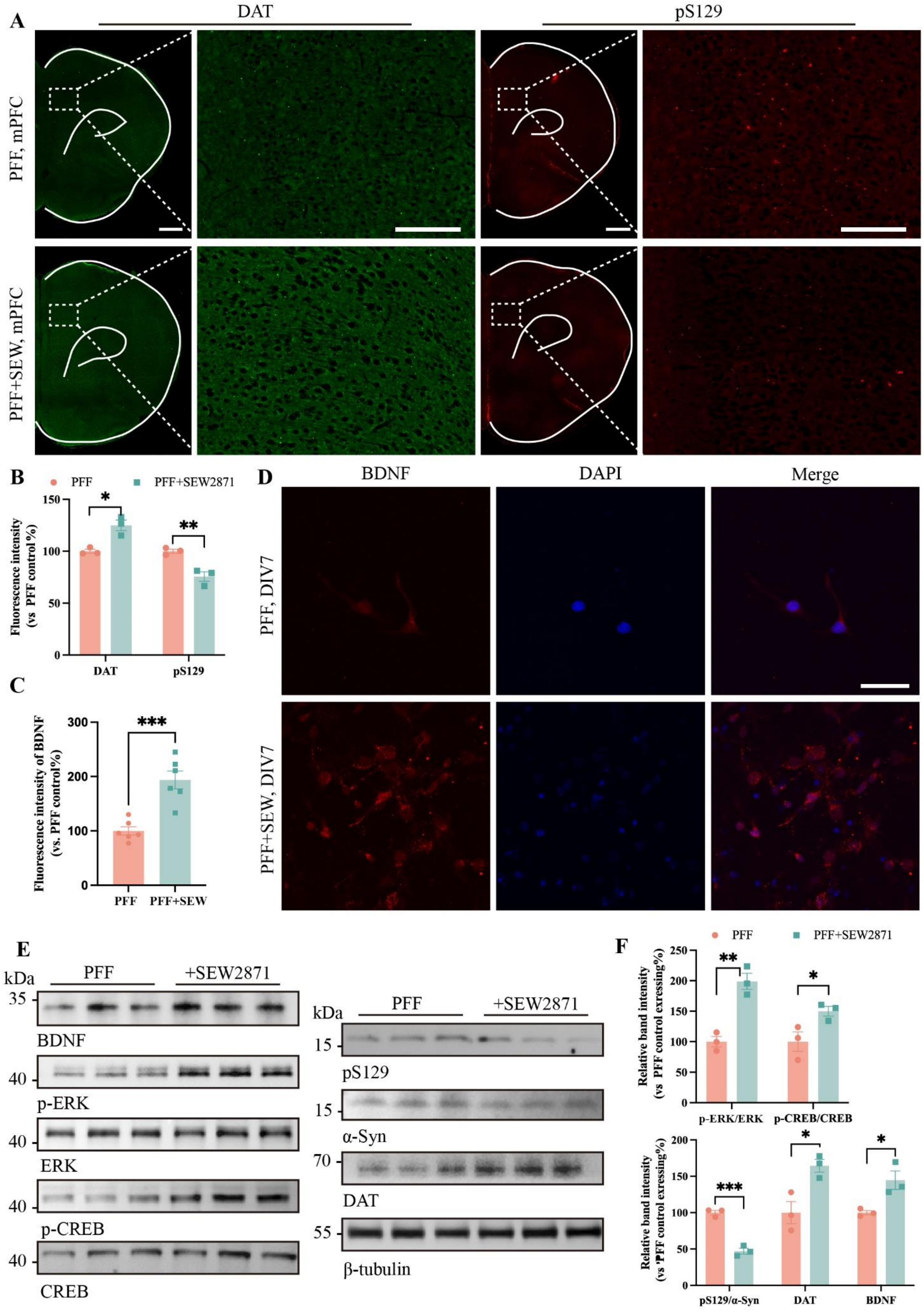

A
DAT
pS129
PFF, mPFC
PFF+SEW, mPFC
B
PFF
PFF+SEW2871
Fluorescence intensity (vs PFF control%)
DAT
pS129
C
Fluorescence intensity of BDNF (vs. PFF control%)
PFF
PFF+SEW
D
BDNF
DAPI
Merge
PFF, DIV7
PFF+SEW, DIV7
E
kDa
PFF
+SEW2871
BDNF
p-ERK
ERK
p-CREB
CREB
pS129
α-Syn
DAT
β-tubulin
F
Relative band intensity (vs PFF control exressing%)
p-ERK/ERK
p-CREB/CREB
pS129/α-Syn
DAT
BDNF

Fig. S6 An ERK agonist improves the PD-like pathology and BDNF content not only *in vivo* but also *in vitro*. **A** DAT is significantly restored, and levels of pathological α-Syn are decreased in the ipsilateral mPFC. Scale bars, 1 mm (left) and 200 μm (right). **D** Significant increase in levels of BDNF in SEW2871-treated neurons. Scale bar, 50 μm. **E** SEW2871 improves the ERK/CREB activation levels and PD-like pathology *in vitro* as *in vivo*. **B**, **C, F** The corresponding statistical analyses. Bars represent the mean, error bars represent the SEM, and symbols represent biologically independent replicates (n). n = 3 in **B** and **F**. n = 6 in **C**. *P <0.05, **P <0.01, ***P <0.001.
